# Solitary Plexiform Neurofibroma in the Urachus Associated With von Recklinghausen’s Disease

**DOI:** 10.7759/cureus.84520

**Published:** 2025-05-21

**Authors:** Hiroshi Ushida, Ryo Ikari

**Affiliations:** 1 Department of Urology, Gakkentoshi Hospital, Kyoto, JPN; 2 Department of Urology, Japan Community Health Care Organization Shiga Hospital, Otsu, JPN

**Keywords:** neurofibromatosis type 1, peripheral nerve sheath tumor, plexiform neurofibroma of the bladder, urachal tumor, von recklinghausen’s disease

## Abstract

Neurofibromatosis type 1 (NF1), also known as von Recklinghausen's disease, is an autosomal dominant disorder characterized by multiple café-au-lait macules and cutaneous neurofibromas. Although neurofibromas are common in NF1, involvement of the urinary tract is rare, with the bladder being the most frequently affected site. Urachal neurofibromas are extremely rare, and their diagnosis and management remain challenging due to nonspecific imaging characteristics and their often asymptomatic presentation.

We report the case of a 30-year-old man with clinical features of NF1 in whom a 5-cm mass at the bladder dome was incidentally identified on MRI performed during evaluation for spinal cord symptoms. Imaging revealed a cone-shaped mass in the urachal region, and cystoscopy showed no mucosal abnormalities. Urinalysis and urine cytology were unremarkable. A transabdominal needle biopsy confirmed the diagnosis of a neurofibroma. Given the tumor’s size (>5 cm) and deep-seated location in the trunk, both known risk factors for malignant transformation, surgical excision was performed, including resection of the urachus and bilateral umbilical ligaments. Histopathological analysis confirmed a plexiform neurofibroma consistent with NF1. The postoperative course was uneventful, with no evidence of recurrence during 36 months of follow-up.

This case highlights the rare presentation of a urachal plexiform neurofibroma in a patient with NF1. To reduce the potential risk of malignant transformation associated with plexiform neurofibromas larger than 5 cm and located in deep trunk regions, accurate diagnosis and timely surgical intervention are essential, even in asymptomatic cases.

## Introduction

Neurofibromatosis (NF) is a rare, autosomal dominant systemic disorder with variable phenotypic expression. NF is classified into three subtypes based on the location of the genetic mutation: type 1 (NF1), type 2 (NF2), and schwannomatosis (SWN) [1,2]. NF1, also known as von Recklinghausen’s disease, is the most common form, with an incidence of one in 2,700 and a prevalence of one in 4,500 [3]. It is characterized by multiple café-au-lait macules and cutaneous neurofibromas [1,2,4].

Neurofibromas are histologically benign peripheral nerve sheath tumors frequently observed in patients with NF1 [1,2,4]. Involvement of the urinary tract is exceedingly rare, with the bladder being the most commonly affected organ [5]. Approximately 70 cases have been reported in the literature [6]. These tumors typically present as diffuse infiltrative lesions and, less commonly, as solitary masses. They are believed to originate from nerves of the pelvic, vesical, and prostatic plexuses [5-7].

We present a rare case of a urachal tumor diagnosed as a solitary plexiform neurofibroma in a patient with NF1. Urachal involvement in NF1 is exceptionally uncommon and unfamiliar to most urologists. Imaging alone posed diagnostic challenges; therefore, a definitive diagnosis of urachal neurofibroma associated with NF1 was made through histopathological analysis of tissue obtained via transabdominal needle biopsy. Although neurofibromas are histologically benign, recent reports have suggested a potential for malignant transformation [4-10], highlighting the importance of careful management strategies, including surgical intervention.

## Case presentation

A 30-year-old man was referred for evaluation after the incidental detection of a mass at the bladder dome on MRI performed at another clinic during assessment for spinal cord symptoms. Physical examination revealed multiple café-au-lait macules and numerous soft, asymmetrically distributed cutaneous nodules. His mother exhibited similar cutaneous findings. Urinalysis and urine cytology were unremarkable.

**Figure 1:**
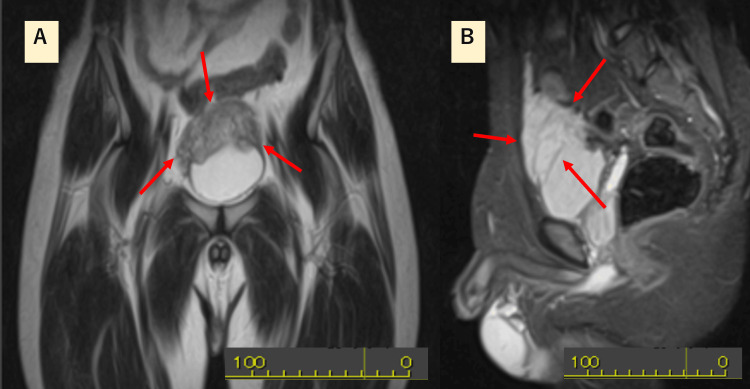
MRI images (A) Coronal pelvic T2-weighted short-tau inversion recovery (T2-STIR) image showing a slightly hypointense, heterogeneous mass; (B) Sagittal pelvic STIR image demonstrating a homogeneously hyperintense mass.

The MRI demonstrated a 5-cm cone-shaped mass in the urachal region displacing the bladder dome. The lesion showed homogeneously low signal intensity on T1-weighted images, homogeneously high signal without a target sign on T2-weighted images, mildly heterogeneous low signal on T2-weighted short-tau inversion recovery (T2-STIR), and homogeneously high signal on STIR (Figures 1A, 1B). 

**Figure 2:**
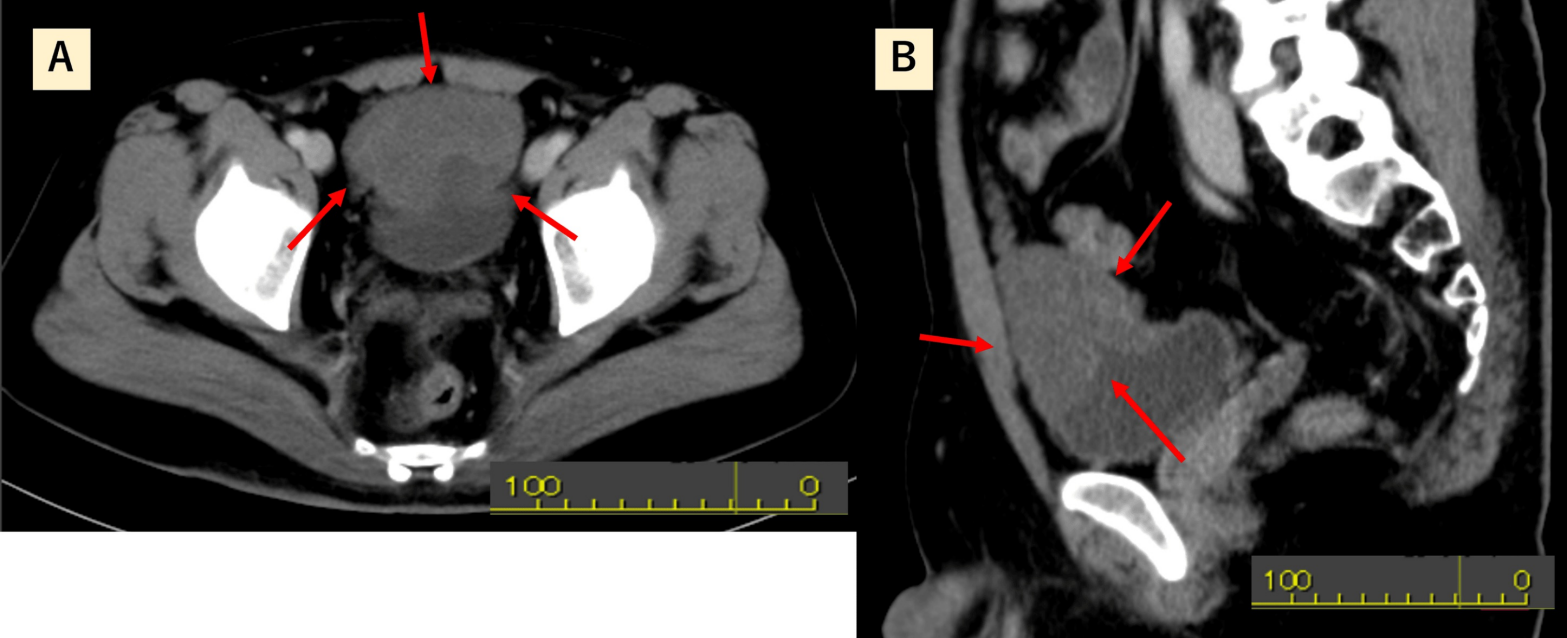
Contrast-enhanced CT images Axial (A) and sagittal (B) abdominal CT scans showing a hypoattenuating mass in the urachal region.

Contrast-enhanced abdominal CT revealed a hypoattenuating mass in the same region (Figures 2A, 2B).

**Figure 3:**
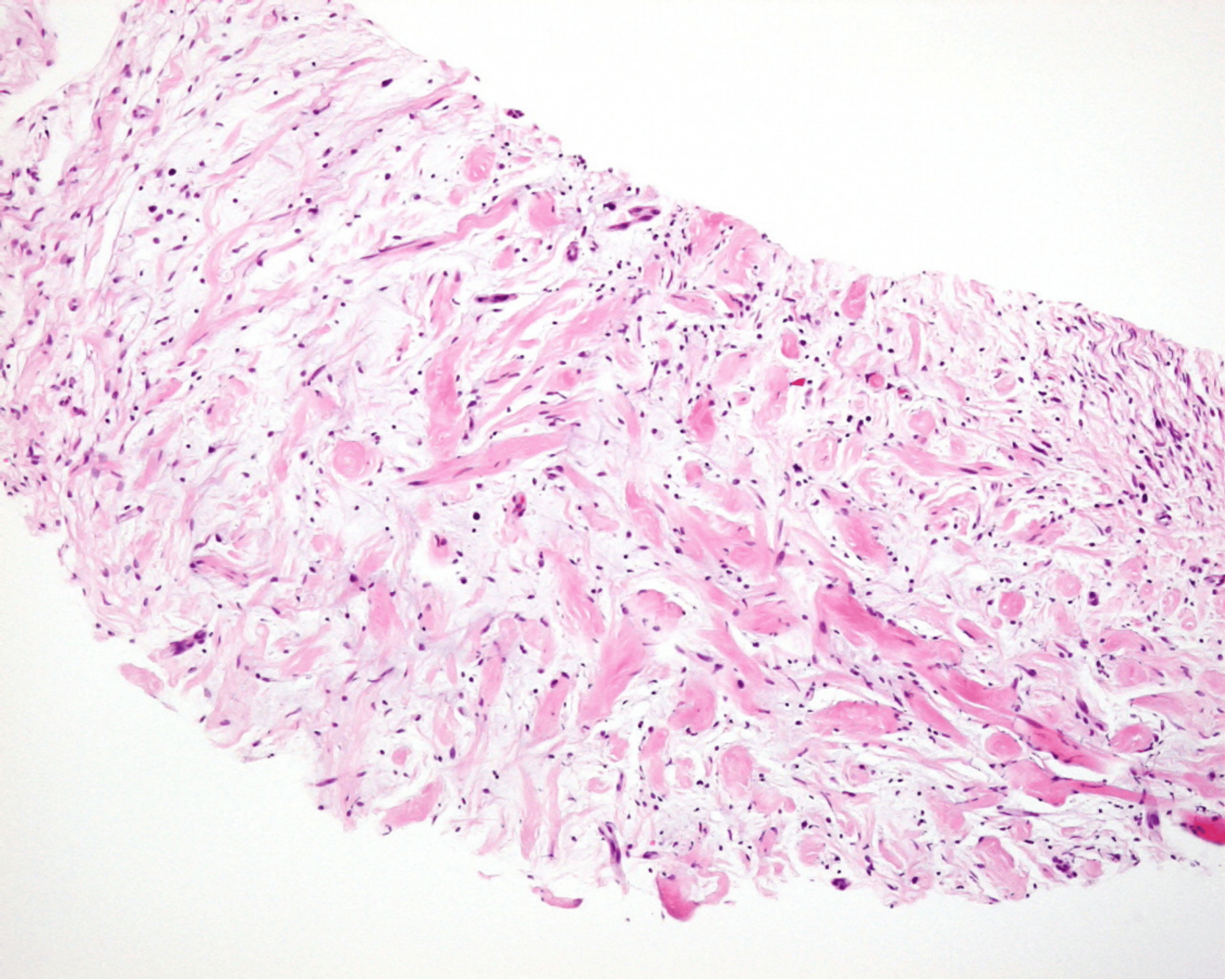
Histopathology of the needle biopsy specimen A hematoxylin and eosin (H&E)-stained section (original magnification ×100) demonstrates a dense proliferation of intersecting nerve fiber bundles.

Cystoscopy revealed intact bladder mucosa despite compression at the dome. Preoperative bladder capacity was approximately 400 mL, with no evidence of urinary dysfunction. Although urachal carcinoma was initially suspected due to the lesion's location, the imaging features were atypical, raising differential diagnoses including submucosal bladder tumor or mesenchymal neoplasm. An ultrasound-guided transabdominal needle biopsy was performed, yielding seven core specimens. Histopathological analysis confirmed a diagnosis of neurofibroma (Figure 3).

**Figure 4:**
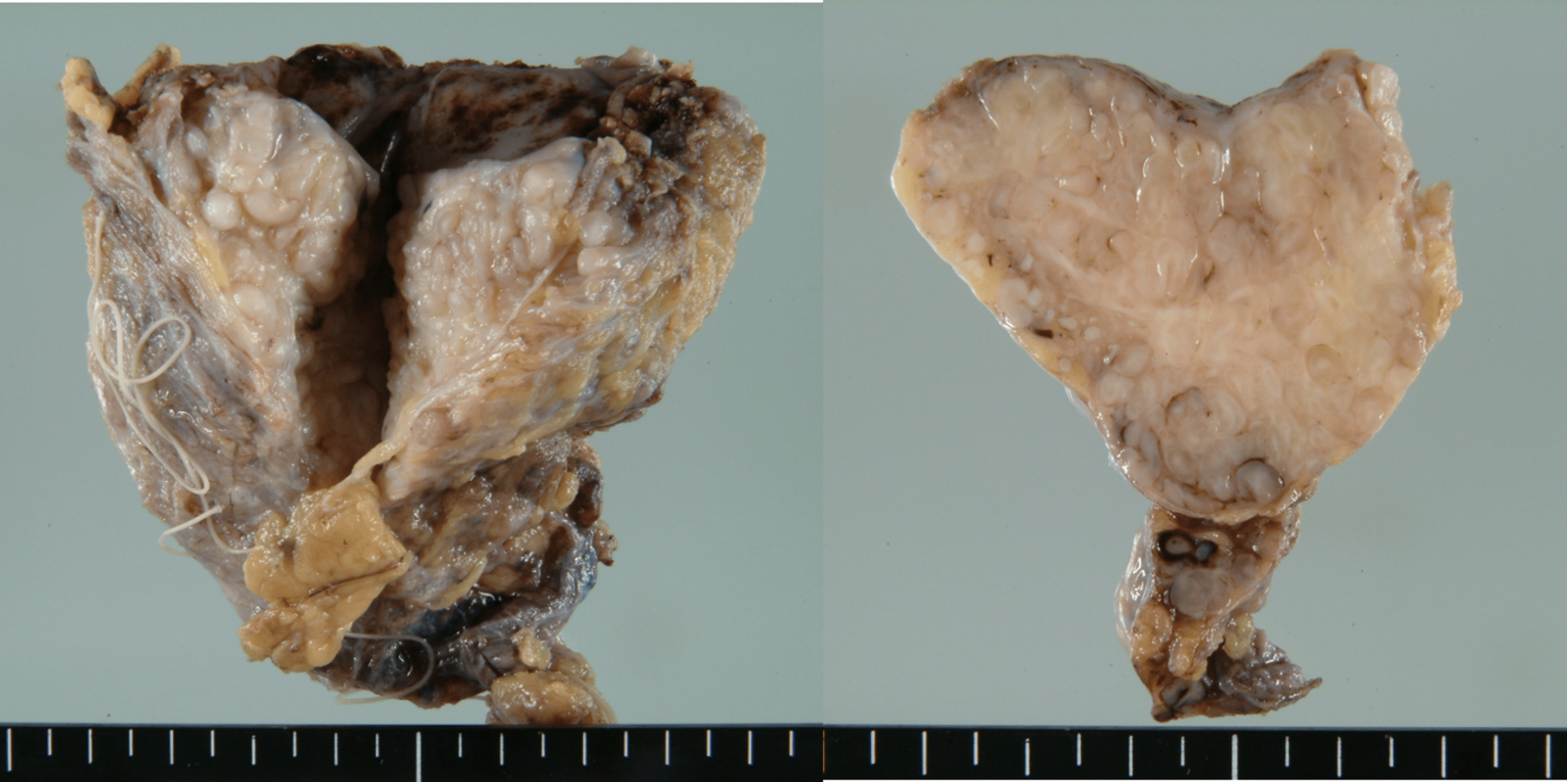
Gross pathology of the resected specimen Macroscopic cross-section revealing multiple nodular formations.

Given the tumor’s size (5 cm) and its deep-seated location in the trunk, both recognized risk factors for malignant transformation, surgical excision was deemed appropriate. The procedure involved en bloc resection of the mass along with the urachus and bilateral umbilical ligaments (Figure 4).

**Figure 5:**
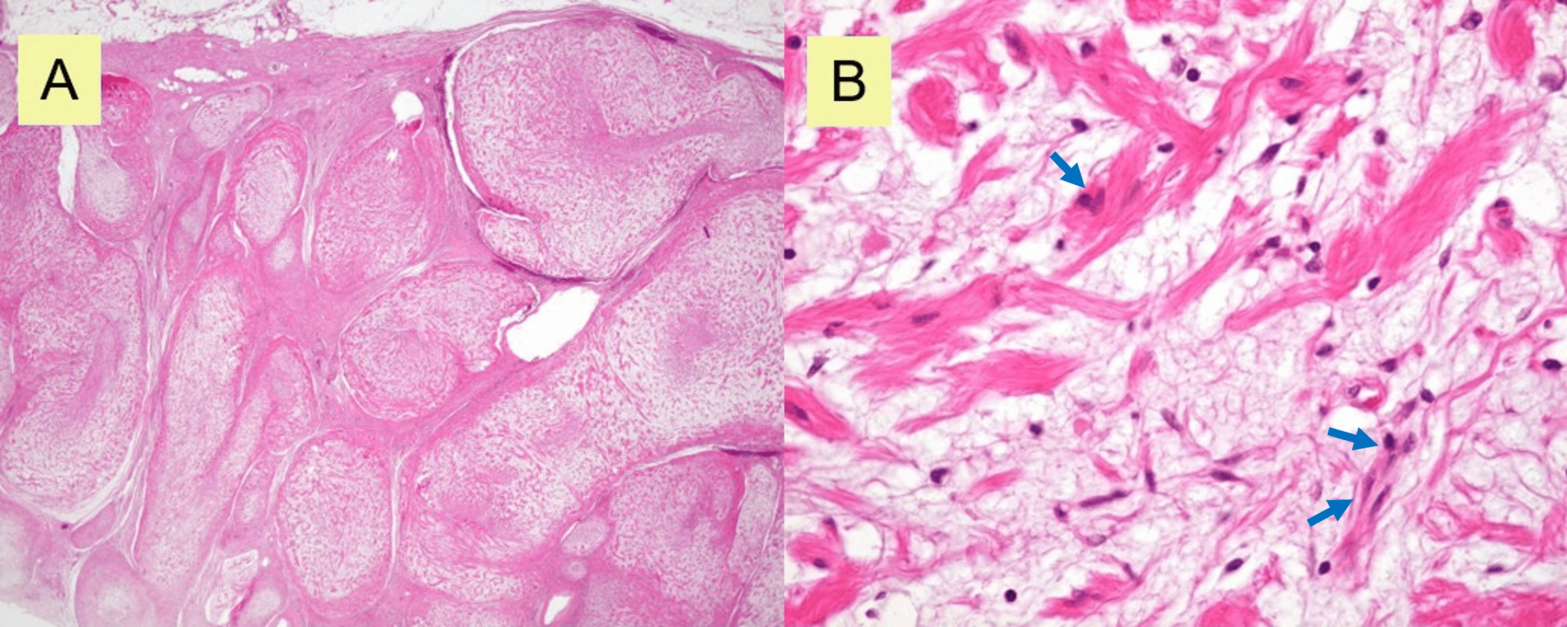
Histopathology of the resected tumor (A) Low-power view (hematoxylin and eosin (H&E) stain, original magnification ×40) demonstrating tortuous enlargement of nerve fiber bundles; (B) High-power view (H&E stain, original magnification ×400) showing spindle-shaped cells with ovoid to elongated nuclei (arrow).

Final histopathological analysis using hematoxylin and eosin (H&E) staining confirmed the diagnosis of plexiform neurofibroma (Figures 5A, 5B), consistent with NF1. Immunohistochemical analysis was not required.

Although postoperative bladder capacity initially decreased to 150 mL, it gradually improved along with the resolution of urinary frequency. The postoperative course was uneventful, and no recurrence was observed during 36 months of follow-up.

## Discussion

NF is classified into three subtypes based on the genetic mutation site: NF1 (peripheral form), NF2 (central form), and SWN [1,2]. NF1 is the most prevalent subtype, accounting for approximately 96% of cases, followed by NF2 (3%) and SWN (<1%) [1,11]. NF1 results from mutations in the NF1 tumor suppressor gene on chromosome 17q11.2 [11-13], with point mutations responsible for about 90% of cases [14,15].

The diagnostic criteria for NF1, originally established by the NIH in 1988 [16], were revised in 2021 to enhance differentiation from conditions such as Legius syndrome and constitutional mismatch repair deficiency (CMMRD) [17]. Our case met three revised criteria under Category A, which were as follows: the presence of six or more café-au-lait macules, axillary/inguinal freckling, and multiple cutaneous neurofibromas predominantly on the trunk, confirming a clinical diagnosis of NF1.

Among genitourinary organs, the bladder is most frequently affected in NF1. Cheng et al. [5] reported fewer than 60 cases of bladder neurofibroma between 1932 and 1999, with Umakanthan et al. [6] later increasing the total to approximately 70. These tumors typically originate from ganglia within the bladder wall, particularly from the vesicoprostatic plexus [5-7]. Two morphological types have been described: solitary and diffuse (plexiform), the latter characterized by infiltration along multiple nerve branches [5,6]. In our case, the tumor likely arose from a nerve bundle within the urachus and extended along the urachal tract.

On MRI, conventional findings of plexiform neurofibromas include low signal intensity on T1-weighted images, high signal intensity on T2-weighted images, and a characteristic “target sign” on sagittal T2-weighted sequences [18]. STIR and T2 fat-suppressed sequences are preferable to CT for non-contrast visualization of these lesions [19]. The presence of a target sign is useful in distinguishing neurofibromas (positive) from malignant peripheral nerve sheath tumors (MPNSTs, typically negative) [20]. In our case, these imaging features were absent, making it difficult to differentiate the lesion from urachal malignancies. The differential diagnosis of urachal tumors includes MPNSTs, leiomyosarcoma, leiomyoma, rhabdomyosarcoma, ganglioneuroma, paraganglioma, fibrosarcoma, fibroma, and inflammatory pseudotumor [5, 6].

Although asymptomatic neurofibromas may be managed conservatively, the lifetime risk of malignant transformation, particularly into MPNSTs, is significant, with reported rates ranging from 8% to 15.8% [4,8,9]. A 20-fold higher incidence of MPNSTs has been observed in individuals with internal plexiform neurofibromas compared to those without them [10]. Tumor size (≥5 cm) and anatomical location, particularly deep-seated sites such as the trunk, have been identified as important risk factors for malignant transformation [21,22]. Therefore, long-term monitoring and timely surgical intervention are essential in the management of NF1-associated neurofibromas [4,8,19].

## Conclusions

We presented a rare case of urachal plexiform neurofibroma in a patient with NF1. Despite the diagnostic limitations of imaging modalities, a definitive diagnosis was achieved through histopathological examination of a needle biopsy specimen. Given the exceptional rarity of urachal involvement in NF1 and the challenges in both diagnosis and management, this case contributes important insights to the limited body of literature and underscores the value of biopsy-guided diagnosis and timely surgical intervention.
